# PARTICIPATION IN TWO EVIDENCE-BASED FALLS PREVENTION PROGRAMS AMONG OLDER ADULTS WITH AND WITHOUT A DISABILITY

**DOI:** 10.1093/geroni/igz038.452

**Published:** 2019-11-08

**Authors:** Thomas J Eagen, Ellen McGough, Tracy Mroz, Deborah Kartin, Anjum Hajat, Ivan Molton

**Affiliations:** University of Washington, Seattle, Washington, United States

## Abstract

Older adults with a disability are at greater risk for falls and injury due to falling compared to those without a disability. Evidence-based falls prevention programs (EBFPPs) have been developed and disseminated broadly, however individuals with disabilities were excluded from original research on effectiveness. Using data from the National Falls Prevention Database from the National Council on Aging, we compared the reach and effectiveness of two EBFPPs, A Matter of Balance (MOB) or Stepping On, between those with and without a disability. Program reach was measured using attendance percentage. Program effectiveness was measured using change in fear of falling (FOF), fall-related activity restriction (FAR), and falls self-efficacy (FSE) post-program. A total of 12,667 participants were analyzed. Participants were, on average, 76 years old (M = 76.18, SD = 9.86), largely female (75%), well educated (80% some college or higher), and white (90%). Nearly half self-reported a disability (40%). Older adults with a disability were as likely to attend (M = 0.88, SD = 0.14) the program compared to those without a disability (M = 0.88, SD = 0.14, p =.30). Older adults with a disability reported greater FOF and FAR and lower FSE compared to participants without a disability at baseline. Significant improvements were made across effectiveness measures, irrespective of disability status. MOB and Stepping On are effective programs, well attended by older adults with and without disabilities, however older adults with a disability continued to report higher FOF and FAR, and lower FSE compared to those without a disability.

